# Absolute configuration of vouaca­pen-5α-ol

**DOI:** 10.1107/S1600536810029557

**Published:** 2010-07-31

**Authors:** Hoong-Kun Fun, Orapun Yodsaoue, Suchada Chantrapromma, Chatchanok Karalai

**Affiliations:** a X-ray Crystallography Unit, School of Physics, Universiti Sains Malaysia, 11800 USM, Penang, Malaysia; b Department of Chemistry, Faculty of Science, Prince of Songkla University, Hat-Yai, Songkhla 90112, Thailand; c Crystal Materials Research Unit, Department of Chemistry, Faculty of Science, Prince of Songkla University, Hat-Yai, Songkhla 90112, Thailand

## Abstract

The title compound, C_20_H_30_O_2_, {systematic name: (4a*R*,6a*S*,7*R*,11a*S*,11b*R*)-4,4,7,11b-tetra­methyl-1,2,3,4,4a,5,6,6a,7,11,11a,11b-dodeca­hydro­phenanthro[3,2-*b*]furan-4a-ol}, is a cas­sane furan­oditerpene which was isolated from the roots of *Caesalpinia pulcherrima*. The absolute configurations at positions 4a, 6a, 7, 11a and 11b are *R*, *S*, *R*, *S* and *R*, respectively. The mol­ecule has four-fused rings consisting of three cyclo­hexane rings and one furan ring. The three cyclo­hexane rings are *trans*-fused. Two cyclo­hexane rings are in chair conformations, while the third is in an envelope conformation. In the crystal structure, the mol­ecules are linked by inter­molecular O—H⋯O hydrogen bonds into a zigzag chain along the *a* axis. A short O⋯O contact [3.0398 (14) Å] is also present.

## Related literature

For ring conformations, see: Cremer & Pople (1975 ▶). For bond-length data, see: Allen *et al.* (1987 ▶). For background to cassane furan­oditerpenes and their biological activities, see: Che *et al.* (1986 ▶); Jiang *et al.* (2001 ▶); McPherson *et al.* (1986 ▶); Promsawan *et al.* (2003 ▶); Ragasa *et al.* (2002 ▶); Smitinand & Larson (2001 ▶); Tewtrakul *et al.* (2003 ▶). For related structures, see: Fun *et al.* (2010 ▶); Jiang *et al.* (2001 ▶). For the stability of the temperature controller used in the data collection, see: Cosier & Glazer (1986 ▶).
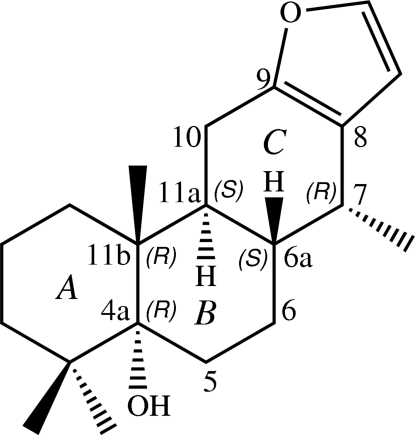


## Experimental

### 

#### Crystal data

C_20_H_30_O_2_
*M* *_r_* = 302.44
Orthorhombic, 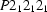

*a* = 6.7367 (2) Å
*b* = 12.7818 (3) Å
*c* = 19.3472 (5) Å
*V* = 1665.93 (8) Å^3^
*Z* = 4
Cu *K*α radiation
μ = 0.58 mm^−1^
*T* = 100 K
0.29 × 0.22 × 0.17 mm

#### Data collection

Bruker APEX DUO CCD area-detector diffractometer
Absorption correction: multi-scan (*SADABS*; Bruker, 2009 ▶) *T*_min_ = 0.851, *T*_max_ = 0.908
30751 measured reflections
2880 independent reflections
2856 reflections with *I* > 2σ(*I*)
*R*_int_ = 0.028

#### Refinement

*R*[*F* ^2^ > 2σ(*F* ^2^)] = 0.027
*wR*(*F* ^2^) = 0.088
*S* = 1.16
2880 reflections
207 parameters
H atoms treated by a mixture of independent and constrained refinement
Δρ_max_ = 0.21 e Å^−3^
Δρ_min_ = −0.25 e Å^−3^
Absolute structure: Flack (1983 ▶), 1202 Friedel pairs
Flack parameter: 0.0 (2)

### 

Data collection: *APEX2* (Bruker, 2009 ▶); cell refinement: *SAINT* (Bruker, 2009 ▶); data reduction: *SAINT*; program(s) used to solve structure: *SHELXTL* (Sheldrick, 2008 ▶); program(s) used to refine structure: *SHELXTL*; molecular graphics: *SHELXTL*; software used to prepare material for publication: *SHELXTL* and *PLATON* (Spek, 2009 ▶).

## Supplementary Material

Crystal structure: contains datablocks global, I. DOI: 10.1107/S1600536810029557/is2581sup1.cif

Structure factors: contains datablocks I. DOI: 10.1107/S1600536810029557/is2581Isup2.hkl

Additional supplementary materials:  crystallographic information; 3D view; checkCIF report

## Figures and Tables

**Table 1:** Hydrogen-bond geometry (Å, °)

*D*—H⋯*A*	*D*—H	H⋯*A*	*D*⋯*A*	*D*—H⋯*A*
O2—H1*O*2⋯O1^i^	0.854 (19)	2.246 (19)	3.0398 (14)	154.7 (18)

Symmetry code: (i) 
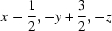
.

**Fig. 1.**
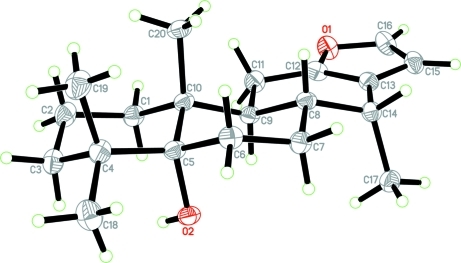
The structure of (I), showing 50% probability displacement ellipsoids and the atom-numbering scheme.

**Fig. 2.**
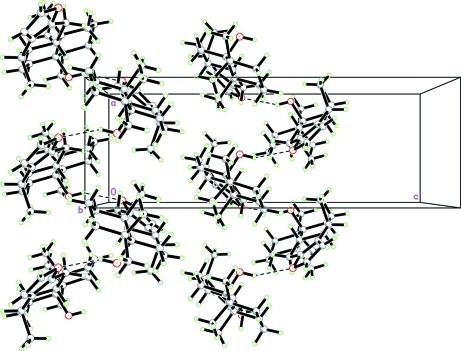
The crystal packing of (I) viewed along the b axis, showing one dimensional chains along the [100]. O—H···O hydrogen bonds are shown as dashed lines.

## supplementary crystallographic information

### Comment

Cassane furanoditerpenes have been found from the plants in the family *Caesalpiniaceae*. The isolated compounds from plants in this family have been reported to show various of bioactivities such as antitumor (Che *et al.*, 1986), antifungal (Ragasa *et al.*, 2002), anti-tubercular (Promsawan *et al.*, 2003), antiviral (Jiang *et al.*, 2001) and HIV-1 protease inhibitory (Tewtrakul *et al.*, 2003) activities. During the course of our research on bioactive compounds from natural-occuring sources, the title cassane furanoditerpene (I) which known as vouacapen-5α-ol (McPherson *et al.*, 1986) was isolated from *Caesalpinia pulcherrima* (L.) Swartz, a small tree which has been used as ornamental (Smitinand & Larson, 2001), abortifacient and emmenagogue purposes. We previously reported the absolute configuration of a cassane furanoditerpene namely isovouacapenol C (Fun *et al.*, 2010) which was isolated from the same plant. Herein the absolute configuration of another cassane furanoditerpene was determined by making use of the anomalous scattering of Cu Kα radiation with the Flack parameter being refined to 0.0 (2) and its crystal structure is reported.

The molecule of the title compound (Fig. 1) is constructed from the fusion of three cyclohexane rings and a furan ring. The three cyclohexane rings which have different conformations are *trans*-fused. Two cyclohexane rings *A* and *B* are in chair conformations whereas the third (ring *C*) adopts an envelope conformation with the puckered C8 atom having the maximum deviation of 0.3012 (14) Å from the best plane of the remaining five atoms (C9/C11–C14) and with the puckering parameters Q =  0.4287 (14) Å and θ = 49.88 (19)° and φ = 5.6 (3)° (Cremer & Pople, 1975). The furan ring (C12/C13/C15/C16/O1) is planar (*rms* 0.0023 (2) Å). The bond distances in (I) are within normal ranges (Allen *et al.*, 1987) and comparable with the related structures which are caesalmin C, D, E, F and G (Jiang *et al.*, 2001) and isovouacapenol C (Fun *et al.*, 2010). The absolute configurations at positions 4a, 6a, 7, 11a and 11b of the vouacapen-5α-ol or atoms C5, C8, C14, C9 and C10 are *R*,*S*,*R*,*S*,*R* configurations.

The crystal packing of (I) is stabilized by intermolecular O—H···O hydrogen bonds (Table 1). The molecules are linked into infinite one dimensional chains along the [100] through O2—H1O2···O1 hydrogen bonds (Fig. 2 and Table 1). O···O [3.0398 (14) Å; symmetry codes -1/2+x, 3/2-y, -z and 1/2+x, 3/2-y, -z] short contacts were observed.

### Experimental

The air-dried roots of *C. pulcherrima* (6.3 kg) were extracted with CH_2_Cl_2_ (2 × 2.5 *L*) for 5 days at room temperature. The combined extracts were concentrated under reduced pressure to afford a dark brownish extract (75.3 g) which was further purified by quick column chromatography (QCC) over silica gel using hexane as eluent and increasing polarity with EtOAc and MeOH to afford 16 fractions (F1-F16). Fraction F2 (5.9 g) was further purified by QCC with hexane-CH_2_Cl_2_ (1:4), yielding the title compound as white solid (50.2 g). Colorless block-shaped single crystals of the title compound suitable for *x*-ray structure determination were recrystallized from CH_2_Cl_2_ by the slow evaporation of the solvent at room temperature after several days (m.p. 371-373 K).

### Refinement

Hydroxy H atoms were located in a difference map and refined isotropically. The remaining H atoms were placed in calculated positions with (C—H) = 0.98 for CH, 0.97 for CH_2_ and 0.96 Å for CH_3_ atoms. The *U*_iso_(H) values were constrained to be 1.5*U*_eq_ of the carrier atom for methyl H atoms and 1.2*U*_eq_ for the remaining H atoms. A rotating group model was used for the methyl groups. The highest residual electron density peak is located at 0.73 Å from C4 and the deepest hole is located at 1.32 Å from C17. 1202 Friedel pairs were used to determine the absolute configuration.

### Crystal data

**Table:**

C_20_H_30_O_2_	*D*_x_ = 1.206 Mg m^−^^3^
*M**_r_* = 302.44	Melting point = 371–373 K
Orthorhombic, *P*2_1_2_1_2_1_	Cu *K*α radiation, λ = 1.54178 Å
Hall symbol: P 2ac 2ab	Cell parameters from 2880 reflections
*a* = 6.7367 (2) Å	θ = 4.6–66.0°
*b* = 12.7818 (3) Å	µ = 0.58 mm^−^^1^
*c* = 19.3472 (5) Å	*T* = 100 K
*V* = 1665.93 (8) Å^3^	Block, colorless
*Z* = 4	0.29 × 0.22 × 0.17 mm
*F*(000) = 664	

### Data collection

**Table:**

Bruker APEX DUO CCD area-detector diffractometer	2880 independent reflections
Radiation source: sealed tube	2856 reflections with *I* > 2σ(*I*)
graphite	*R*_int_ = 0.028
φ and ω scans	θ_max_ = 66.0°, θ_min_ = 4.6°
Absorption correction: multi-scan (*SADABS*; Bruker, 2009)	*h* = −7→7
*T*_min_ = 0.851, *T*_max_ = 0.908	*k* = −15→15
30751 measured reflections	*l* = −22→22

### Refinement

**Table:**

Refinement on *F*^2^	Secondary atom site location: difference Fourier map
Least-squares matrix: full	Hydrogen site location: inferred from neighbouring sites
*R*[*F*^2^ > 2σ(*F*^2^)] = 0.027	H atoms treated by a mixture of independent and constrained refinement
*wR*(*F*^2^) = 0.088	*w* = 1/[σ^2^(*F*_o_^2^) + (0.0545*P*)^2^ + 0.2119*P*] where *P* = (*F*_o_^2^ + 2*F*_c_^2^)/3
*S* = 1.16	(Δ/σ)_max_ = 0.001
2880 reflections	Δρ_max_ = 0.21 e Å^−^^3^
207 parameters	Δρ_min_ = −0.25 e Å^−^^3^
0 restraints	Absolute structure: Flack (1983), 1202 Friedel pairs
Primary atom site location: structure-invariant direct methods	Flack parameter: 0.0 (2)

### Special details

**Table:**

Experimental. The crystal was placed in the cold stream of an Oxford Cryosystems Cobra open-flow nitrogen cryostat (Cosier & Glazer, 1986) operating at 100.0 (1) K.
Geometry. All esds (except the esd in the dihedral angle between two l.s. planes) are estimated using the full covariance matrix. The cell esds are taken into account individually in the estimation of esds in distances, angles and torsion angles; correlations between esds in cell parameters are only used when they are defined by crystal symmetry. An approximate (isotropic) treatment of cell esds is used for estimating esds involving l.s. planes.
Refinement. Refinement of F^2^ against ALL reflections. The weighted R-factor wR and goodness of fit S are based on F^2^, conventional R-factors R are based on F, with F set to zero for negative F^2^. The threshold expression of F^2^ > 2sigma(F^2^) is used only for calculating R-factors(gt) etc. and is not relevant to the choice of reflections for refinement. R-factors based on F^2^ are statistically about twice as large as those based on F, and R- factors based on ALL data will be even larger.

### Fractional atomic coordinates and isotropic or equivalent isotropic displacement parameters (Å^2^)

**Table:**

	*x*	*y*	*z*	*U*_iso_*/*U*_eq_	
O1	1.03886 (16)	0.52792 (8)	0.07346 (5)	0.0334 (2)	
O2	0.57276 (13)	0.96871 (7)	0.08320 (5)	0.0258 (2)	
C1	0.9773 (2)	0.93648 (11)	0.02533 (7)	0.0275 (3)	
H1A	1.0886	0.8924	0.0127	0.033*	
H1B	0.8677	0.9195	−0.0052	0.033*	
C2	1.0353 (2)	1.05118 (11)	0.01446 (8)	0.0328 (3)	
H2A	1.1552	1.0662	0.0404	0.039*	
H2B	1.0636	1.0628	−0.0341	0.039*	
C3	0.8709 (2)	1.12537 (11)	0.03751 (8)	0.0304 (3)	
H3A	0.7575	1.1163	0.0072	0.036*	
H3B	0.9172	1.1968	0.0323	0.036*	
C4	0.8027 (2)	1.10897 (11)	0.11288 (7)	0.0273 (3)	
C5	0.7496 (2)	0.98994 (10)	0.12352 (7)	0.0235 (3)	
C6	0.6857 (2)	0.96538 (11)	0.19790 (7)	0.0265 (3)	
H6A	0.7961	0.9786	0.2289	0.032*	
H6B	0.5779	1.0116	0.2109	0.032*	
C7	0.6185 (2)	0.85208 (11)	0.20597 (7)	0.0264 (3)	
H7A	0.4932	0.8434	0.1819	0.032*	
H7B	0.5948	0.8385	0.2546	0.032*	
C8	0.76551 (19)	0.77021 (11)	0.17865 (6)	0.0235 (3)	
H8A	0.8849	0.7742	0.2075	0.028*	
C9	0.8275 (2)	0.79830 (10)	0.10381 (6)	0.0231 (3)	
H9A	0.7059	0.7988	0.0760	0.028*	
C10	0.91614 (19)	0.91155 (10)	0.10034 (7)	0.0233 (3)	
C11	0.9692 (2)	0.71734 (11)	0.07021 (7)	0.0295 (3)	
H11A	0.9471	0.7157	0.0207	0.035*	
H11B	1.1058	0.7381	0.0783	0.035*	
C12	0.9355 (2)	0.61239 (11)	0.09923 (7)	0.0274 (3)	
C13	0.8133 (2)	0.58241 (11)	0.15075 (7)	0.0261 (3)	
C14	0.6792 (2)	0.65857 (11)	0.18679 (6)	0.0249 (3)	
H14A	0.6797	0.6413	0.2362	0.030*	
C15	0.8399 (2)	0.47142 (12)	0.15815 (7)	0.0316 (3)	
H15A	0.7751	0.4278	0.1893	0.038*	
C16	0.9764 (3)	0.44313 (11)	0.11136 (7)	0.0349 (3)	
H16A	1.0227	0.3752	0.1053	0.042*	
C17	0.4653 (2)	0.64563 (12)	0.16019 (7)	0.0301 (3)	
H17A	0.4312	0.5727	0.1595	0.045*	
H17B	0.4555	0.6738	0.1143	0.045*	
H17C	0.3758	0.6824	0.1902	0.045*	
C18	0.6175 (2)	1.17717 (12)	0.12459 (8)	0.0370 (4)	
H18A	0.6459	1.2480	0.1113	0.056*	
H18B	0.5815	1.1753	0.1726	0.056*	
H18C	0.5097	1.1508	0.0972	0.056*	
C19	0.9636 (3)	1.14968 (12)	0.16241 (8)	0.0376 (4)	
H19A	0.9689	1.2246	0.1600	0.056*	
H19B	1.0900	1.1211	0.1494	0.056*	
H19C	0.9324	1.1287	0.2088	0.056*	
C20	1.1033 (2)	0.91693 (12)	0.14665 (8)	0.0308 (3)	
H20A	1.1679	0.8500	0.1470	0.046*	
H20B	1.0654	0.9354	0.1929	0.046*	
H20C	1.1928	0.9688	0.1288	0.046*	
H1O2	0.602 (3)	0.9757 (14)	0.0405 (10)	0.038 (5)*	

### Atomic displacement parameters (Å^2^)

**Table:**

	*U*^11^	*U*^22^	*U*^33^	*U*^12^	*U*^13^	*U*^23^
O1	0.0389 (6)	0.0318 (5)	0.0295 (5)	0.0068 (5)	0.0075 (4)	0.0007 (4)
O2	0.0193 (5)	0.0341 (5)	0.0239 (5)	−0.0007 (4)	−0.0017 (4)	0.0012 (4)
C1	0.0247 (7)	0.0333 (7)	0.0245 (7)	−0.0005 (6)	0.0057 (5)	0.0007 (5)
C2	0.0313 (8)	0.0389 (8)	0.0283 (7)	−0.0058 (6)	0.0075 (6)	0.0034 (6)
C3	0.0345 (8)	0.0288 (7)	0.0278 (7)	−0.0061 (6)	0.0020 (6)	0.0016 (5)
C4	0.0290 (7)	0.0296 (7)	0.0232 (7)	−0.0039 (6)	0.0000 (6)	−0.0007 (5)
C5	0.0192 (6)	0.0302 (7)	0.0212 (6)	−0.0002 (5)	0.0002 (5)	0.0000 (5)
C6	0.0258 (6)	0.0328 (7)	0.0210 (6)	0.0017 (6)	0.0025 (5)	−0.0028 (5)
C7	0.0242 (6)	0.0342 (7)	0.0207 (6)	0.0013 (6)	0.0058 (5)	0.0015 (5)
C8	0.0195 (6)	0.0324 (7)	0.0184 (6)	0.0010 (6)	−0.0001 (5)	0.0016 (5)
C9	0.0192 (6)	0.0306 (7)	0.0196 (6)	0.0015 (5)	0.0015 (5)	0.0009 (5)
C10	0.0184 (6)	0.0299 (7)	0.0216 (6)	−0.0009 (5)	0.0005 (5)	0.0004 (5)
C11	0.0285 (7)	0.0334 (7)	0.0267 (7)	0.0032 (6)	0.0075 (6)	0.0019 (6)
C12	0.0284 (7)	0.0314 (7)	0.0224 (7)	0.0064 (6)	0.0001 (5)	−0.0029 (5)
C13	0.0265 (7)	0.0306 (7)	0.0213 (6)	−0.0006 (5)	−0.0032 (5)	0.0011 (5)
C14	0.0248 (6)	0.0330 (7)	0.0169 (6)	−0.0005 (6)	0.0019 (5)	0.0020 (5)
C15	0.0380 (8)	0.0323 (7)	0.0244 (7)	−0.0020 (7)	−0.0005 (6)	0.0029 (6)
C16	0.0480 (9)	0.0277 (7)	0.0290 (7)	0.0059 (7)	0.0004 (7)	0.0022 (6)
C17	0.0267 (7)	0.0376 (7)	0.0260 (7)	−0.0036 (6)	0.0012 (6)	0.0025 (6)
C18	0.0438 (9)	0.0311 (7)	0.0363 (8)	0.0042 (7)	0.0063 (7)	−0.0006 (6)
C19	0.0437 (9)	0.0375 (8)	0.0315 (8)	−0.0123 (7)	−0.0046 (7)	−0.0035 (6)
C20	0.0191 (6)	0.0412 (8)	0.0321 (7)	−0.0023 (6)	−0.0034 (6)	0.0025 (6)

### Geometric parameters (Å, °)

**Table:**

O1—C16	1.3745 (17)	C9—C11	1.5507 (18)
O1—C12	1.3780 (16)	C9—C10	1.5673 (18)
O2—C5	1.4494 (16)	C9—H9A	0.9800
O2—H1O2	0.85 (2)	C10—C20	1.5486 (18)
C1—C2	1.5319 (19)	C11—C12	1.4719 (19)
C1—C10	1.5417 (18)	C11—H11A	0.9700
C1—H1A	0.9700	C11—H11B	0.9700
C1—H1B	0.9700	C12—C13	1.348 (2)
C2—C3	1.525 (2)	C13—C15	1.437 (2)
C2—H2A	0.9700	C13—C14	1.5001 (19)
C2—H2B	0.9700	C14—C17	1.5388 (19)
C3—C4	1.5432 (19)	C14—H14A	0.9800
C3—H3A	0.9700	C15—C16	1.340 (2)
C3—H3B	0.9700	C15—H15A	0.9300
C4—C19	1.538 (2)	C16—H16A	0.9300
C4—C18	1.538 (2)	C17—H17A	0.9600
C4—C5	1.5764 (19)	C17—H17B	0.9600
C5—C6	1.5345 (18)	C17—H17C	0.9600
C5—C10	1.5699 (18)	C18—H18A	0.9600
C6—C7	1.525 (2)	C18—H18B	0.9600
C6—H6A	0.9700	C18—H18C	0.9600
C6—H6B	0.9700	C19—H19A	0.9600
C7—C8	1.5347 (18)	C19—H19B	0.9600
C7—H7A	0.9700	C19—H19C	0.9600
C7—H7B	0.9700	C20—H20A	0.9600
C8—C14	1.5489 (19)	C20—H20B	0.9600
C8—C9	1.5493 (17)	C20—H20C	0.9600
C8—H8A	0.9800		
			
C16—O1—C12	105.67 (11)	C10—C9—H9A	106.8
C5—O2—H1O2	108.0 (13)	C1—C10—C20	108.54 (11)
C2—C1—C10	113.28 (11)	C1—C10—C9	109.45 (10)
C2—C1—H1A	108.9	C20—C10—C9	109.06 (11)
C10—C1—H1A	108.9	C1—C10—C5	109.14 (10)
C2—C1—H1B	108.9	C20—C10—C5	112.86 (11)
C10—C1—H1B	108.9	C9—C10—C5	107.75 (10)
H1A—C1—H1B	107.7	C12—C11—C9	110.69 (11)
C3—C2—C1	111.68 (11)	C12—C11—H11A	109.5
C3—C2—H2A	109.3	C9—C11—H11A	109.5
C1—C2—H2A	109.3	C12—C11—H11B	109.5
C3—C2—H2B	109.3	C9—C11—H11B	109.5
C1—C2—H2B	109.3	H11A—C11—H11B	108.1
H2A—C2—H2B	107.9	C13—C12—O1	110.69 (12)
C2—C3—C4	114.09 (12)	C13—C12—C11	129.43 (12)
C2—C3—H3A	108.7	O1—C12—C11	119.88 (12)
C4—C3—H3A	108.7	C12—C13—C15	106.14 (13)
C2—C3—H3B	108.7	C12—C13—C14	121.80 (12)
C4—C3—H3B	108.7	C15—C13—C14	132.02 (13)
H3A—C3—H3B	107.6	C13—C14—C17	109.80 (11)
C19—C4—C18	106.74 (12)	C13—C14—C8	108.93 (11)
C19—C4—C3	109.45 (12)	C17—C14—C8	114.61 (11)
C18—C4—C3	107.68 (12)	C13—C14—H14A	107.8
C19—C4—C5	113.90 (11)	C17—C14—H14A	107.8
C18—C4—C5	110.10 (11)	C8—C14—H14A	107.8
C3—C4—C5	108.79 (11)	C16—C15—C13	106.54 (13)
O2—C5—C6	103.66 (10)	C16—C15—H15A	126.7
O2—C5—C10	108.31 (10)	C13—C15—H15A	126.7
C6—C5—C10	109.74 (10)	C15—C16—O1	110.95 (13)
O2—C5—C4	107.27 (10)	C15—C16—H16A	124.5
C6—C5—C4	112.55 (11)	O1—C16—H16A	124.5
C10—C5—C4	114.61 (10)	C14—C17—H17A	109.5
C7—C6—C5	111.92 (11)	C14—C17—H17B	109.5
C7—C6—H6A	109.2	H17A—C17—H17B	109.5
C5—C6—H6A	109.2	C14—C17—H17C	109.5
C7—C6—H6B	109.2	H17A—C17—H17C	109.5
C5—C6—H6B	109.2	H17B—C17—H17C	109.5
H6A—C6—H6B	107.9	C4—C18—H18A	109.5
C6—C7—C8	114.87 (11)	C4—C18—H18B	109.5
C6—C7—H7A	108.5	H18A—C18—H18B	109.5
C8—C7—H7A	108.5	C4—C18—H18C	109.5
C6—C7—H7B	108.5	H18A—C18—H18C	109.5
C8—C7—H7B	108.5	H18B—C18—H18C	109.5
H7A—C7—H7B	107.5	C4—C19—H19A	109.5
C7—C8—C14	110.54 (11)	C4—C19—H19B	109.5
C7—C8—C9	109.74 (11)	H19A—C19—H19B	109.5
C14—C8—C9	114.19 (11)	C4—C19—H19C	109.5
C7—C8—H8A	107.4	H19A—C19—H19C	109.5
C14—C8—H8A	107.4	H19B—C19—H19C	109.5
C9—C8—H8A	107.4	C10—C20—H20A	109.5
C8—C9—C11	113.77 (11)	C10—C20—H20B	109.5
C8—C9—C10	110.90 (10)	H20A—C20—H20B	109.5
C11—C9—C10	111.33 (10)	C10—C20—H20C	109.5
C8—C9—H9A	106.8	H20A—C20—H20C	109.5
C11—C9—H9A	106.8	H20B—C20—H20C	109.5
			
C10—C1—C2—C3	−55.24 (17)	C11—C9—C10—C5	170.45 (11)
C1—C2—C3—C4	55.00 (16)	O2—C5—C10—C1	66.93 (13)
C2—C3—C4—C19	72.73 (15)	C6—C5—C10—C1	179.45 (11)
C2—C3—C4—C18	−171.60 (12)	C4—C5—C10—C1	−52.77 (14)
C2—C3—C4—C5	−52.30 (15)	O2—C5—C10—C20	−172.30 (10)
C19—C4—C5—O2	169.30 (11)	C6—C5—C10—C20	−59.78 (14)
C18—C4—C5—O2	49.46 (14)	C4—C5—C10—C20	68.00 (14)
C3—C4—C5—O2	−68.32 (13)	O2—C5—C10—C9	−51.83 (13)
C19—C4—C5—C6	55.92 (15)	C6—C5—C10—C9	60.69 (13)
C18—C4—C5—C6	−63.92 (15)	C4—C5—C10—C9	−171.54 (10)
C3—C4—C5—C6	178.30 (11)	C8—C9—C11—C12	28.58 (16)
C19—C4—C5—C10	−70.41 (15)	C10—C9—C11—C12	154.78 (11)
C18—C4—C5—C10	169.75 (11)	C16—O1—C12—C13	−0.18 (16)
C3—C4—C5—C10	51.97 (14)	C16—O1—C12—C11	179.32 (13)
O2—C5—C6—C7	59.70 (13)	C9—C11—C12—C13	−4.3 (2)
C10—C5—C6—C7	−55.82 (14)	C9—C11—C12—O1	176.33 (12)
C4—C5—C6—C7	175.28 (11)	O1—C12—C13—C15	−0.23 (16)
C5—C6—C7—C8	51.72 (15)	C11—C12—C13—C15	−179.67 (15)
C6—C7—C8—C14	−177.79 (11)	O1—C12—C13—C14	−178.36 (11)
C6—C7—C8—C9	−50.97 (15)	C11—C12—C13—C14	2.2 (2)
C7—C8—C9—C11	−177.42 (11)	C12—C13—C14—C17	102.74 (15)
C14—C8—C9—C11	−52.68 (15)	C15—C13—C14—C17	−74.84 (18)
C7—C8—C9—C10	56.15 (14)	C12—C13—C14—C8	−23.52 (17)
C14—C8—C9—C10	−179.10 (10)	C15—C13—C14—C8	158.90 (14)
C2—C1—C10—C20	−70.08 (15)	C7—C8—C14—C13	172.14 (10)
C2—C1—C10—C9	171.00 (11)	C9—C8—C14—C13	47.82 (14)
C2—C1—C10—C5	53.30 (15)	C7—C8—C14—C17	48.70 (14)
C8—C9—C10—C1	179.65 (11)	C9—C8—C14—C17	−75.62 (14)
C11—C9—C10—C1	51.88 (14)	C12—C13—C15—C16	0.56 (17)
C8—C9—C10—C20	61.04 (13)	C14—C13—C15—C16	178.42 (14)
C11—C9—C10—C20	−66.72 (13)	C13—C15—C16—O1	−0.70 (17)
C8—C9—C10—C5	−61.79 (13)	C12—O1—C16—C15	0.56 (17)

### Hydrogen-bond geometry (Å, °)

**Table:**

*D*—H···*A*	*D*—H	H···*A*	*D*···*A*	*D*—H···*A*
O2—H1O2···O1^i^	0.854 (19)	2.246 (19)	3.0398 (14)	154.7 (18)

Symmetry codes: (i) *x*−1/2, −*y*+3/2, −*z*.
